# A Falls Prevention Program for People After Stroke in Guyana: An International Collaboration

**DOI:** 10.1093/ptj/pzae107

**Published:** 2024-08-07

**Authors:** Maureen Romanow Pascal, Barbara Lawrence, Stephanie Pires, Elton Newton, Deoranie Babulall, Kelly Saroka, Megan Shaver, Mackenzie Schanzlin, Kristi Pearage

**Affiliations:** Physical Therapy Department, Misericordia University, Dallas, Pennsylvania, USA; Palms Rehabilitation Department, Ministry of Health, c/o Palms Geriatric Home, Brickdam, Georgetown, Guyana; Palms Rehabilitation Department, Ministry of Health, c/o Palms Geriatric Home, Brickdam, Georgetown, Guyana; Palms Rehabilitation Department, Ministry of Health, c/o Palms Geriatric Home, Brickdam, Georgetown, Guyana; Palms Rehabilitation Department, Ministry of Health, c/o Palms Geriatric Home, Brickdam, Georgetown, Guyana; Physical Therapy Department, Misericordia University, Dallas, Pennsylvania, USA; Physical Therapy Department, Misericordia University, Dallas, Pennsylvania, USA; Physical Therapy Department, Misericordia University, Dallas, Pennsylvania, USA; Physical Therapy Department, Misericordia University, Dallas, Pennsylvania, USA

**Keywords:** Balance, Cerebrovascular Accident, Gait, Mobility, Rehabilitation, Stroke, Walking

## Abstract

**Objective:**

The objective was to describe the social, environmental, and cultural adaptations to an existing falls program and assess acceptability and preliminary effectiveness of the program in reducing fear, reducing falls, and improving function among individuals poststroke in Guyana.

**Methods:**

A quasi-experimental pilot study with a pretest/posttest in-group design was developed through a collaboration of researchers in Guyana and the US. Participants took part in the falls prevention program for 8 weeks. Outcome measures included a 10-m walk test, the Five Times Sit to Stand Test, and subjective questionnaires for falls incidence and balance confidence at the beginning and end.

**Results:**

Twenty participants completed the study. One participant experienced medical complications, and their data were excluded from analysis. Fifteen participants (78.9%) demonstrated improvements in comfortable and fast walking speed. Twelve participants completed the Five Times Sit to Stand Test. Eleven (91.67%) improved their time at the posttest, with 9 (81.8%) demonstrating a clinically important improvement. Nineteen participants had sustained at least 1 fall prior to the study. Only 1 participant reported a fall during the program. Initially, the majority of participants (11/19) were very concerned about falling. At the end, only 1 was very concerned about falling, and the majority (15/19) were not concerned at all. Posttest surveys of participants indicated acceptability of the program.

**Conclusions:**

This pilot program helped reduce fall risk and improve confidence, gait speed, and community mobility of the study participants. Future research at other rehabilitation departments in Guyana would help increase the generalizability of the program.

**Impact:**

The program can be used clinically by physical therapists in Guyana, both in departments and as a home program. Shared knowledge and experience of researchers considering research evidence and the environmental, social, and economic conditions of people living in Guyana were important in developing an effective program.

## Introduction

Stroke is the chief cause of death and disability worldwide, with more than 12 million people experiencing a stroke each year.^1^ While falls are common in any population, individuals who have had a stroke are at a much greater risk for falls. After a stroke, it is common to see impaired balance, changes in gait pattern, poor functional mobility, and a subsequent fear of falling. Due to these potentially long-term impacts, the incidence of falls in individuals poststroke is between 1.3 and 6.5 falls in the first year after the stroke.^2^ Those who experience a fall after a stroke can have severe consequences, including head trauma, fractures, a severe decline in activities of daily living, and decreased activity participation, as well as decreased confidence and a fear of falling.^3^

The incidence of strokes and falls varies worldwide, with those in low- and middle-income countries disproportionately affected. According to the World Health Organization (WHO), over 80% of fall-related fatalities occur in low- and middle-income countries.^4^ Although Guyana is currently classified by the World Bank as having an upper-middle income economy with a gross national annual income of $6,600 per capita,^5^ at least 75% of individuals in Guyana are classified as poor.^6^ The stress of poverty contributes to the inverse, disparate relationship between socioeconomic status and the prevalence of hypertension and type 2 diabetes mellitus in Guyana,^7–10^ with those who are poorer being more likely to develop these non-communicable diseases. Hypertension and diabetes are risk factors for stroke; similarly, stroke disproportionately affects the poor in Guyana. A study by Sockalingam et al noted that stroke was the leading cause of death in the country in 2017.^9^ Stroke and ischemic heart disease are leading causes of disability in Guyana starting at age 50.^10^ Guyana is considered part of the “Global Stroke Belt”^11^ and has one of the highest rates of disability-adjusted life years lost due to stroke worldwide.

Falls are the second leading cause of unintentional death and injury throughout the world, resulting in costly medical expenses and premature loss of independence.^1^ Falls pose a major problem in every part of the world and create a public health situation that requires immediate and frequent attention. Individuals in Guyana who have experienced difficulty recovering their function poststroke are at an even greater risk for falls, associated injury, and further loss of the ability to participate in previous life roles.

Inequitable distribution of social, economic, and environmental resources contributes to disparate outcomes for individuals following stroke. Those with limited socioeconomic resources are less able to access rehabilitation services; this same group is disproportionately exposed to unsafe conditions in the natural and built environment. Because those with least resources have the greatest incidence of risk factors for stroke, more environmental challenges, and more difficulty accessing rehabilitation services, these individuals are most likely to experience disability after a fall poststroke. In Guyana, the majority of the population and economic activity is centered around the low-lying coastal area of Georgetown, the capital of the country. Georgetown is located along the Atlantic coast of South America and sits below sea level, making the city prone to flooding and causing many environmental hazards within the community.^6^^,^^12^^,^^13^ Frequent flooding has resulted in many potholes and uneven walking surfaces in cities and surrounding communities. A recent land survey found unpaved roads throughout 73% of the neighborhood of Sophia, one of the poorest neighborhoods near Georgetown.^13^ Many individuals poststroke in Guyana struggle to function within their community due to the fall risk imposed by similar conditions. Home environments may also predispose individuals with limited income to falls. Due to flooding, the living space of many homes is on the second floor, meaning stair navigation is needed to enter or exit the home. Most homes are not accessible to wheelchairs or walkers, with narrow doorways to enter the bathroom or between rooms and small living quarters. Most people do not have access to equipment, such as bed rails, grab bars, or raised toilet seats, to improve accessibility, increase independence, and decrease fall risk. Due to inaccessible home environments, almost all people who return home after a stroke perform some walking, with the assist of family members as needed. Although assisted walking can promote independence, home conditions and inadequate training of family can lead to falls and reluctance to walk unless necessary. It is common for individuals poststroke to tell physical therapists they limit their activity at home and in the community after a fall, fearful of another fall and associated injuries.

Guyana has a universal healthcare system. Care is provided free of charge at hospitals and at all rehabilitation departments. However, since public services are limited in scope and availability, those who can afford them may seek care at a private hospital or medical clinic. There are only about 50 practicing physical therapists in Guyana, which averages to about 0.62 therapist/10,000 people.^14^ In order to maximize the number of people who can receive services, physical therapists work closely with rehabilitation assistants to provide care. Rehabilitation assistants receive training about techniques used in physiotherapy, occupational therapy, and speech therapy and guide people through many aspects of their rehabilitation program. Most people return home approximately 1 week after having a stroke and complete the majority of their therapy in an outpatient rehabilitation department. The Palms Rehabilitation Department is the primary rehabilitation department for Region 4, which includes Georgetown and the surrounding area, a population of about 350,000 people, and stroke is the most common diagnosis treated. A small number of people in Guyana pay for services at a private hospital or private clinic. People who need rehabilitation often must travel a distance to the nearest rehabilitation department. Many individuals are unable to reach the departments due to a lack of transportation. Public transportation using the bus system of minibuses is difficult for many as the buses are often very crowded and are not accessible for those in a wheelchair or unable to negotiate stairs. Private taxi services are available but are not affordable for most people to use on a regular basis.

When the rehabilitation staff at Palms Rehab (physical therapists, an occupational therapist, and rehabilitation assistants) recognized the need for a falls prevention program for people poststroke, the lead physical therapist at Palms Rehab (B.L.) contacted a faculty member (M.P.) at Misericordia University. The two have worked together since 2007 to provide exchanges of cultural and educational ideas and experiences between therapists and students from Guyana and the United States. After our initial communication, we began our collaboration between the rehabilitation staff at Palms Rehab and physical therapy students and faculty from Misericordia University. We modeled our pilot project on the WHO Rehabilitation 2030 strategy.^15^ As part of our situation assessment, we held multiple virtual meetings. The team in Guyana provided background information to the US team, discussing the need for a program tailored to the needs of the poststroke population that addressed the specific environmental and geographical factors commonly contributing to falls in Guyana. We endeavored to develop an evidence-based falls prevention program addressing these factors. We determined it would be important to include people who walk with the assist of a family member at home, as well as those who are more independent but at risk for falls in the community, as these 2 groups encompass the majority of individuals poststroke who seek services in the rehabilitation departments.

Due to staffing and physical space constraints, the group decided the program at Palms Rehab should be offered via a combination of individual and group sessions. In an unpublished collaborative effort in 2017, 3 of the researchers (B.L., D.B., and M.P.) successfully implemented a group exercise program for people poststroke in the Georgetown area. The success of that program, which has persisted and even grown in membership despite the pandemic, provided insight into the cultural acceptance of group exercise. Members of the rehabilitation department at Palms Rehab have remarked that the members of the group exercise program have developed a support system, regularly checking in on each other and sharing stories via a WhatsApp group. This development is especially important since a history of heart attack, angina, or stroke were associated with a high risk of suicidal behaviors in Guyana.^16^ Since regular exercise and social support are both known to be important interventions to improve mental health, it was agreed group exercise sessions were a valuable part of the proposed program.

The team in the US conducted a literature review and found that while there is a large body of evidence suggesting that the implementation of a falls prevention program can be effective in decreasing the incidence and fear of falls, the majority of studies focused on the elderly population without neurological diagnoses.^17–23^ Common interventions leading to the success of these programs include physical exercise, home modifications, balance training, walking, lower extremity strengthening, increased knowledge of community hazards, and fall prevention strategies. Of the studies that included patients with neurological conditions,^24–27^ only one focused exclusively on people poststroke.^27^ All studies were completed in high resource countries, which may limit their generalizability to the home and outdoor conditions in Guyana. Although numerous evidence-based falls prevention programs exist, the cultural and social acceptability of these programs, and the feasibility and effectiveness of their implementation in Guyana are not known.

The objectives of this pilot study are to: (1) describe the social, environmental, and cultural adaptations to an existing falls program; and (2) assess the acceptability and preliminary effectiveness of the program in reducing fear, reducing falls, and improving function among individuals poststroke in Guyana. We hypothesize that the falls prevention program created will be acceptable to clinicians and people who use it, and that completing the exercise program for 8 weeks will help to decrease both fall risk and fear of falling in individuals poststroke.

## Methods

### Study Design

This was a quasi-experimental pilot study using a within-group pretest-posttest design.

### Participants

Participants with a history of stroke who were receiving rehabilitation services at the Palms Rehabilitation Department, an outpatient rehabilitation center located in Georgetown, Guyana, were recruited for the study. Inclusion criteria consisted of a history of stroke; a history of at least one fall since their stroke, or fall risk (scoring less than 50 points on the Berg Balance Scale,^28^ or less than 22 points on the Functional Gait Assessment^29^); and the ability to follow simple directions. Exclusion criteria consisted of a history of other neurological disorders such as Alzheimer disease or Parkinson disease to focus specifically on persons poststroke, as this is the most common neuromuscular diagnosis. Other comorbidities, such as type 2 diabetes or cardiovascular disease, were not excluded due to the high prevalence of these comorbidities. Participants did not need to be able to walk in order to participate.

Twenty eligible participants were recruited through convenience sampling via verbal recruitment or through handouts at Palms Rehab from May through August 2022. Participant ages ranged from 45 to 89 years old, and time since stroke ranged from 2 weeks to 10 years (Tab. 1). All participants provided written informed consent prior to participation. There were no potential participants who were excluded from the study; all 20 original potential participants met the inclusion criteria.

**Table 1:** Participant Demographics and Fall History—Pretest Survey

Age (y)	N	Time Since Stroke	N	# of Falls Since Stroke	N	Fall Location	N	Falls Limiting Activities?	N
45–55	5	2 wk	3	0	1	Inside home	14	Yes	11
55–65	3	1–2 mo	2	1	7	Outside home	1	No	8
65–70	7	3–6 mo	6	2	5	Inside and outside	3	Yes/no	1
70–80	3	7–15 mo	6	3–9	6	Hospital	1		
80–90	1	> 3 y	3	10	1	No falls	1		
No answer	1								
Total	20		20		20		20		20

### Intervention

Participants attended one session per week lasting one hour in duration. All interventions were guided by the therapy staff (physical therapists, occupational therapists, or rehabilitation assistants) currently working at Palms Rehab. They worked in both individual and group settings to complete an exercise program designed to target common deficits seen poststroke. Participants also received a copy of the program to use at home in an attempt to increase frequency of exercises (see Suppl. Material 1).

The CoDuSe (Core Stability, Dual-Task, Sensory Strategies) program developed by Forsberg and Nilsagård^27^ provided an excellent starting point for exercises and photos that could easily be modified to include materials that would be available in Guyana and attainable by those we hoped would perform the exercises at home. The program targeted multiple areas of balance by including therapeutic exercises and activities with a focus on static and dynamic balance. There was also a focus on functional activities to improve daily tasks and safety, which fit well with the goals of our program. Exercises in the original program, such as carrying a tray and getting on and off the floor, simulated indoor activities involved in cooking and cleaning. These activities were modified only slightly in our program, using objects from Guyana, and photos that more resembled the program participants. The original program included exercises in supine. These were not included in our program to help emphasize the importance of activity by limiting the time on a treatment mat in the clinic or a bed at home. The CoDuSe program included exercises with eyes closed to promote the use of sensory strategies. These exercises were deemed important to promote balance in the dark. Due to its proximity to the equator, twilight occurs at approximately 6:00 pm year-round, meaning any evening activities out of the home require navigation of uneven surfaces in the dark. Frequent power outages also necessitate the practice of indoor mobility in the dark. Dual-task high-intensity balance and strengthening interventions were provided for all participants, as well as ambulation interventions for those able to walk. To ensure participant safety, exercises were performed in parallel bars as needed, or next to a treatment mat or a wall with close guarding from trained staff. Assistive devices were used as needed during walking exercises. After a group discussion, we added T’ai Chi exercises to our program based on research completed by Koh et al^26^ to encourage standing weight-shifting activities.

### Outcome Measures

Demographic information, fall history, and fear of falling were determined via a questionnaire developed by the physical therapists in Guyana (Tab. 1). The questionnaire was administered verbally and included questions about whether the participant had experienced a fall since their stroke, the circumstances around the fall (ie, where the fall occurred), and how concerned the participant was about future falls (see Suppl. Material 2). The group had discussed using the Short Falls Efficacy Scale International (FESI) but did not use it in favor of one question about the degree of concern for falling, using the same scale as the FESI. The clinician researchers in Guyana expressed concern about the amount of time it would take to complete the questionnaire and thus opted for a single question.

The primary outcome measures used for pretest and posttest measures were the 10 Meter Walk Test (10MWT) (both comfortable and fast walking speed), the Five Times Sit to Stand (FTSTS), and the questionnaire developed by the physical therapists in Guyana. The 10MWT and the FTSTS can assess improvements in lower extremity strength and endurance, functional mobility, and gait when repeated over time.^30–32^ The 10MWT has an excellent reliability coefficient (coefficient ≥ 0.903) and intra- and inter-rater reliability (ICC ≥ 0.87) in the adult and children populations with neurological diseases,^33^^,^^34^ The 10MWT has been shown to be predictive of community ambulation in older adults and adults poststroke.^30^ The FTSTS also demonstrates excellent test–retest reliability (ICC = 0.994) and interrater reliability (ICC 0.970).^35^

The questionnaire was used to assess the frequency of falls and fear of falling in participants throughout the study.

At the first session, each participant completed the FTSTS. Participants who were able to walk completed 3 trials of the 10MWT at their self-selected speed, followed by 3 trials at their fastest gait speed, taking breaks as needed. All measures were repeated at the last session. The same researcher (SP) completed the pretest and posttest measures at Palms Rehab. (Tabs. 1–4).

### Data Collection and Management

All data was collected by the research team at Palms Rehab in Guyana. The data was stored at Palms Rehab on a device that only the researchers could access. The data was then deidentified prior to being analyzed at Misericordia University.

### Data Analysis

Each participant’s data was analyzed separately, using the percent change in scores for the FTSTS and the comfortable and fast speeds of the 10MWT. Changes were compared to the minimal clinically important differences for the 10MWT, and the minimal detectable change (MDC) for the 10MWT and FTSTS. Pretest and posttest information from the questionnaire were tabulated by question and compared.

Due to our small sample size, the Wilcoxon signed-rank test was used to analyze the pretest/posttest differences for all outcome measures.

### Role of the Funding Source

The funders played no role in the design, conduct, or reporting of this study.

## Results

### Preliminary Effectiveness

All 20 participants completed the 8-week program. However, one participant’s data was excluded from the overall analysis due to the development of a more serious medical condition.

All participants completed the 10MWT for comfortable and fast walking speeds (Tab. 2). Improvement in comfortable walking speed on the 10MWT was seen in 15 of 19 participants (79.0%), with 3 participants demonstrating a minimal clinically important difference^36^ and 5 demonstrating a MDC.^37^ For fast walking speed, 16 of 19 participants (84.2%) showed improvement, with 5 demonstrating an MCID and 7 demonstrating a MDC.

**Table 2:** Ten-Meter Walk Test and Self-Reported Gait Status*^a^*

	10MWT—Comfortable Walking Speed (m/s)			10MWT—Fast Walking Speed (m/s)			Self-Reported Gait Status at End of Study
Participant	Pretest	Posttest	Change	Pretest	Posttest	Change	
1	0.14	0.08	0.06	0.14	0.09	0.05	Household
8	0.18	0.21	−0.02	0.33	0.34	0.01	Household
12	0.42	0.46	−0.04	0.78	0.97	−0.19*^b^**^,^**^c^*	Household
15	0.51	0.43	0.09	0.62	0.56	0.06	Household
18	0.20	0.24	−0.04	0.26	0.32	−0.06	Household
9	0.61	0.65	−0.04*^b^*	0.74	0.83	−0.09	Community with family member
2	0.38	0.40	−0.02	0.61	0.64	−0.03	Community with family member
11	0.61	0.65	−0.04	0.70	0.84	−0.14*^c^*	Community with family member
16	0.98	1.02	−0.05	1.12	1.19	−0.07	Community with family member
20	0.94	1.04	−0.09	1.31	1.30	0.01	Community with family member
19	0.55	0.98	−0.43*^b^**^,^**^c^*	0.88	1.24	−0.36*^b^**^,^**^c^*	Community with family member
5	0.38	0.52	−0.14*^c^*	0.74	0.79	−0.05	Independent in community
4	0.28	0.25	0.03	0.30	0.32	−0.02	Independent in community
17	0.56	0.61	−0.05	0.89	0.92	−0.02	Independent in community
7	0.10	1.00	−0.90*^b^**^,^**^c^*	0.15	1.20	−1.06*^b^**^,^**^c^*	Independent in community
10	0.86	0.83	0.03	1.18	1.32	−0.14*^c^*	Independent in community
13	0.16	0.43	−0.27*^b^**^,^**^c^*	0.29	0.48	−0.19*^b^**^,^**^c^*	Independent in community
14	1.36	1.48	−0.12	1.75	1.78	−0.04	Independent in community
3	0.49	0.56	−0.07	0.73	0.85	−0.12*^c^*	Independent in community

^a^ Change in negative indicates improved speed. 10MWT = 10-meter walk test.

^b^ Minimal Clinically Important Difference (MCID) of at least 0.16 m/s.

^c^ Minimal Detectable Change (MDC): acute stroke: 0.11 m/s; chronic stroke, comfortable: 0.18 m/s, fast: 0.13 m/s.

Because 8 participants were unable to complete a sit-to-stand transfer independently, only 12 participants completed the FTSTS (Tab. 3). For the FTSTS, 11 of 12 participants (91.7%) improved their time, nine of which (81.8%) demonstrated a MDC.^38^ Improvements in time to complete the FTSTS ranged from 3.58 to 37.6 seconds faster than pretest scores.

**Table 3:** Five Times Sit to Stand

Participant	Pretest (s)	Posttest (s)	Change in s	% Change
8	23.67	18.22	5.45*^a^*	23.0%
12	17.42	12.64	4.78*^a^*	27.4%
9	23.07	15.39	7.68*^a^*	33.3%
16	6.37	6.09	0.28	4.4%
20	14.23	16.1	−1.87	−13.1%
19	19.63	10.08	9.55*^a^*	48.7%
5	61.35	23.89	37.46*^a^*	61.1%
17	19.34	12.48	6.86*^a^*	35.5%
7	25.17	17.04	8.13*^a^*	32.3%
10	13.4	9.82	3.58*^a^*	26.7%
13	43.08	36.26	6.82*^a^*	15.8%
14	9.44	9.13	0.31	3.3%

^a^ Minimal Detectable Change (MDC) of at least 2.4 s.

The pretest and posttest questionnaires asked participants to rate their fear of falling on a 4-point scale ranging from “Very Concerned” to “Not Concerned at All.” One participant did not answer the pretest question about fear of falling, selecting “I don’t know.” At the pretest, 53% of respondents (10/19) were very concerned about falling. At the posttest, only 16% (3/19) reported they were very concerned about falling, and 74% (14/19) reported they were not very concerned about falls (Figure).

**Figure:**
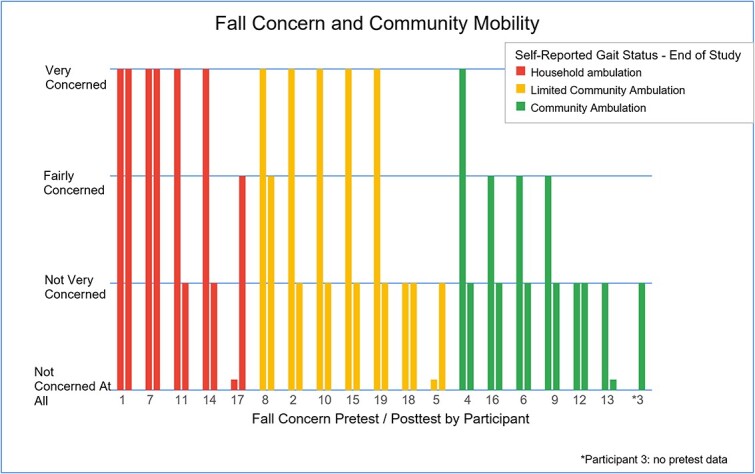
Participants grouped by independence in community in relation to concern of falling. Those who were less concerned about falling tended to have more community mobility, while those concerned about falling tended to only walk at home.

The posttest questionnaire also asked participants about their ability to walk in the community, including whether they were able to walk alone or required a family member with them (Tab. 4). 26% (5/19) reported they walked at home only, while the remaining reported some community mobility, evenly split between walking alone and walking with a family member (Figure). All data was analyzed using the Wilcoxon signed-rank test^39^ in Microsoft Excel. There was a statistically significant difference between the pretest and posttest for all measures (Tab. 5).

**Table 4:** Posttest Survey: Fear of Falling, Activity, and Exercise

**Participant**	**Fall Concern**	**Fear of Falls Limiting Community Activity**	**Return to Daily Activities**	**Walking in Community**	**Continued With Exercise Program**
1	Very	Yes	No	No	No*^a^*
2	Not very	No	Yes	Yes	Yes
3	Not at all	No	Yes	Yes	Yes
4	Not very	Yes	Yes	Yes	Yes
5	Not very	No	Yes	Yes	Yes
7	Not very	No	Yes	Yes	Yes
8	Very	Yes	No	No	Yes
9	Fairly	Yes	Yes	Yes	No*^a^*
10	Not at all	No	Yes	Yes	Yes
11	Not very	No	Yes	Yes	Yes
12	Not very	No	Yes	No	Yes
13	Not very	No	Yes	Yes	Yes
14	Not at all	No	Yes	Yes	Yes
15	Not very	Yes	Yes	No	Yes
16	Not very	No	Yes	Yes	Yes
17	Not very	No	Yes	Yes	No*^a^*
18	Fairly	Yes	Yes	No	Yes
19	Not very	No	Yes	Yes	Yes
20	Not very	No	Yes	Yes	Yes

^a^ Reasons for not continuing exercise program: Participant 1: diagnosed with cancer after study and stopped exercising; Participant 9: traveling out of country; and Participant 17: no support.

**Table 5:** Pretest/Posttest Analyses*^a^*

**Wilcoxon Signed Rank Test Results**	**Sample Size**	**Smaller Sum**	**Critical Value** ^39^	*P*
Comfortable walking speed	19	31	46	*P* < .05*^b^*
Fast walking speed	19	19	46	*P* < .05*^b^*
FTSTS	12	3	13	*P* < .05*^b^*
Concern about falling	14	17.5	21	*P* < .05*^b^*
Fall frequency	18	0	40	*P* < .05*^b^*

^a^ FTSTS = Five Times Sit to Stand.

^b^ Statistically significant.

### Acceptability

The exercise program meets many of the criteria for acceptability as described by Sekhon et al.^40^ These include affective attitude, burden, self-efficacy, perceived effectiveness, intervention coherence, and ethicality. Affective attitude was assessed using the posttest questionnaire. All participants reported enjoying the exercise program, indicating acceptability. At a 2-week follow-up, 16 participants reported they were continuing with the home exercise program, indicating acceptable burden (perceived effort) and self-efficacy. Clinician burden was addressed by the combination of group and individual exercise sessions. Clinician self-efficacy and intervention coherence affected which exercises ended up being used most consistently. Clinicians met regularly with one of the researchers (SP) to discuss the program. Based on these meetings, the program was modified as needed in the overall program and for individual participants. Although the group had agreed to the addition of T’ai Chi exercises, clinicians did not feel comfortable demonstrating the exercises, and so they were regularly excluded. Although 6 people continued to report a fear of falls limiting their daily activities, 18 reported they were able to participate in more of their daily activities since starting the program. This provides an indication that the program has perceived effectiveness. Based on our collaborative approach, and our consideration of the need to adapt exercises based on social, cultural, and environmental resources, we believe this program is acceptable from an ethical perspective.

## Discussion

Through this project, we sought to describe the social, environmental, and cultural adaptations to an existing falls program and to assess the acceptability and preliminary effectiveness of the program in reducing fear, reducing falls, and improving function among individuals poststroke in Guyana. Our findings suggest that the modified falls program developed through our collaborative process demonstrates both acceptability and preliminary effectiveness.

The results indicate preliminary effectiveness and provide foundational evidence for the benefit of using a falls prevention program to improve function and confidence as well as minimize the incidence of falls in a population of individuals who have had a stroke in Guyana. As has been shown in many other studies, there was a positive relationship between confidence in community mobility and gait speed.^30–32^ Over the 8-week program, the majority of participants demonstrated an improvement in function and a decrease in fear of falling, as demonstrated by the subjective report and results of the FTSTS and the 10MWT. Of interest is the weak relationship among participant age, time since stroke, and improvement. Our small sample size with wide ranges in age, time since stroke, and function among the participants likely play a factor in this weak observation. The study included 2 groups of participants: those who could walk and those who were unable to walk unassisted. Since these 2 groups are representative of the population of individuals poststroke who are commonly treated at Palms Rehab and in other rehabilitation departments in Guyana, we were able to assess the preliminary effectiveness and acceptability of the program for both groups.

The development of an acceptable program required collaboration and communication. We felt it was important to consider the social, environmental, and cultural resources in Guyana prior to implementing a program that was developed elsewhere. The long-term relationship developed between the Rehabilitation Department of the Ministry of Health in Guyana and faculty and students at Misericordia University was instrumental in this collaboration, which has been strengthened by a sharing of ideas and a willingness of both groups to learn about the values and culture of the other. The recognition of multiple disparities, such as environmental conditions and access to transportation, as we developed the program was important. Since there are multiple factors that increase a person’s risk for falls after a stroke, it was important to consider and prioritize interventions to maximize activity and participation.^3^^,^^17^^,^^18^^,^^25–27^ The inclusion of a home exercise component was especially important due to the difficulty for many participants to access transportation. Due to uneven walking surfaces throughout the country, including walking and dual-task activities was an important part of the program to improve balance confidence. Future research will likely need to focus on modifying or expanding the home exercise program. The majority of the population lives in rural areas, with limited access to emergency and rehabilitation care.^12^ Although the Ministry of Health employs physical therapists in all regions of the country, the landscape of the country and the low population density still limit the number of people who can access rehabilitation services on a regular basis. For this reason, an exercise program that can be performed at home shortly after its introduction may help improve poststroke mobility in multiple regions of the country.

### Limitations

Our convenient, purposive sample size of 19 participants is small, limiting generalizability of the results at this time. Because this was an unblinded study, there was the potential for bias during data collection by the researchers. The research team used consistent procedures and anonymity to maximize uniformity, but both were difficult at times since the research occurred as part of the work day at Palms Rehab. Because participants became familiar with the staff, there is also the potential for bias as they answered subjective questions about their progress and mobility. These subjective questionnaires were developed as part of this study and have not been validated. The pretest questionnaire did not include a question related to community mobility, which prevented a more thorough pretest–posttest comparison. Future studies will need to include a consideration of the best way to obtain the necessary subjective data, either by revision of the questionnaire used in this study, or compiling other available forms. We used only two outcome measures (FTSTS and 10MWT) for objective data collection. These measures were chosen because they are part of the clinical practice guidelines for assessing balance, gait, and transfers poststroke.^41^ However, since participants performed sit-to-stand activities and walking during the exercise program, the improvement in these measures may be related to their practice. The use of additional measures may have provided more information, but was determined not to be feasible due to time constraints.

### Program Sustainability and Future Research

We have taken some early steps and continue to plan to promote sustainability. The first step we have taken is to share the program with another rehabilitation department, and the physical therapist there has agreed to start using it. The researchers plan to continue to share the program with other regions of the country. In the WHO Rehabilitation 2030 strategy,^15^ Phase 2 is the development of a strategic plan. It is the hope of the researchers that we will continue to collaborate and work toward developing a strategic plan to reduce falls and improve community mobility for many of the individuals in Guyana who have sustained a stroke. The Ministry of Health requires most clinicians to rotate their assignment location approximately every 2 years. Some of the physical therapists and rehabilitation assistants who assisted in the implementation of the program at Palms Rehab have been transferred to other regions, providing an opportunity for them to share the program there. Making this shared implementation part of a strategic plan could be an important step in sustainability. The plan would also likely include training of clinicians and a plan to obtain the balance equipment used in the falls prevention program for other rehabilitation departments.

Future research with a larger sample size could help discern if there are certain parts of the program, such as T’ai Chi or sit-to-stand exercises while holding an item, that are more beneficial than others depending on stroke chronicity, severity, independence in gait, and age. The use of a more comprehensive outcome measure such as the Functional Gait Assessment may provide better insight about how the exercise program may improve the activities required for safe community mobility. Future research and collaboration could also focus on the use of telerehabilitation as one possible strategy to increase access of the program and/or the frequency with which it is performed.

## Conclusions

The goal of this study was to establish a program that would provide physical therapists in Guyana with evidence of the efficacy of a falls prevention program for individuals poststroke. Based on our results, a falls prevention program tailored toward functional activities while considering geographical and environmental factors can be effective in decreasing both fall risk and fear of falling in this population.

The long-term collaboration of research entities in this project facilitated discussion, cooperation, and feedback. We recognize the importance of developing a relationship between partners, considering reality and needs, recognizing and celebrating expertise, and embracing cultural humility. We believe the positive results we observed would have been difficult to achieve and would be less sustainable without this respectful relationship and commitment.^42–44^

## Supplementary Material

2023-0439_R2_Supplementary_Material_1_pzae107

2023-0439_R2_Supplementary_Material_2_pzae107

## Data Availability

The data that support the findings of this study are available upon reasonable request from the corresponding author (MP). The data are not publicly available due to their containing information that could compromise the privacy of research participants.
