# Supplementary material for: A Falls Prevention Program for People After Stroke in Guyana: An International Collaboration
Source: Phys Ther. 2024 Aug 7;104(10):pzae107. doi: 10.1093/ptj/pzae107 (PMC11523612; doi:10.1093/ptj/pzae107)

Supplementary Material 1: FALLS PREVENTION EXERCISE PROGRAMME

-adapted from : Nilsagård Y, von Koch L, Nilsson M, Forsberg A. Balance exercise program reduced falls in people with multiple sclerosis: a single group pretest, posttest trial. Archives of Physical Medicine and Rehabilitation, e-pub 6 jul 2014. Used with permission.

Exercises are divided into four groups:

1) Exercises in Quadruped / Hands and Knees
2) Dual-Task Exercises
3) Sensory Strategies
4) T'ai Chi exercises

"The exercises are designed to improve transfers from the floor, improve strength, and improve functional balance. Most exercises should be performed for at least 30 seconds, if possible. If a patient is unable to tolerate 30 seconds, start with less time and try to build up to more time. Try to minimize the amount of rest between activities, to maximize the time spent working. Some patients may need more or longer rest breaks."[1]

GROUP 1: EXCERCISES ON HANDS AND KNEES (QUADRUPED):

1. Tighten your abdominal (core) muscles by trying to move your navel towards the ceiling. Try to hold for 30 seconds. Rest for 10-20 seconds.

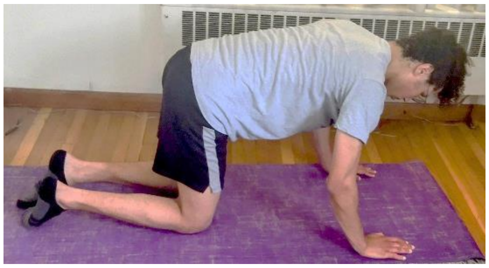

2. Rock forward, so there is more weight on your arms than your legs. Try to put equal weight through both arms. Try to hold this position for 30 seconds. Rest for 10-20 seconds.

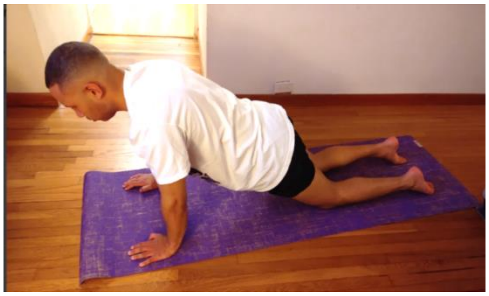

Modified from: ©A Forsberg, Y Nilsagård. This programme was developed in a research study, Group balance training for persons with multiple sclerosis". Reference. Nilsagård Y, von Koch L, Nilsson M, Forsberg A. Balance exercise program reduced falls in people with multiple sclerosis: a single group pretest, posttest trial. Archives of Physical Medicine and Rehabilitation, e-pub 6 juli 2014. Used with permission.

3. Crawl forward; once you reach the edge of the mat, crawl backwards. Repeat as many times as possible in 30 seconds. Rest for 10-20 seconds.

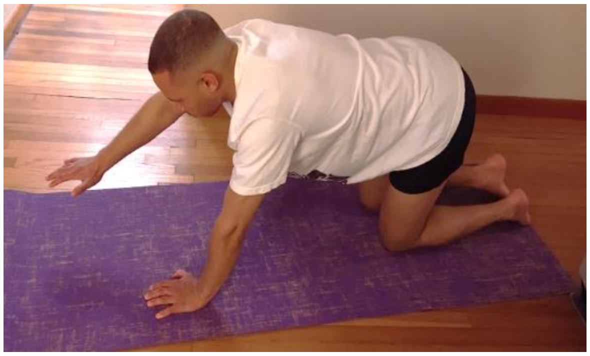

4. Put both hands on therapy ball. Try to put equal weight through both arms. Try to hold this position for 30 seconds. Rest for 10-20 seconds.

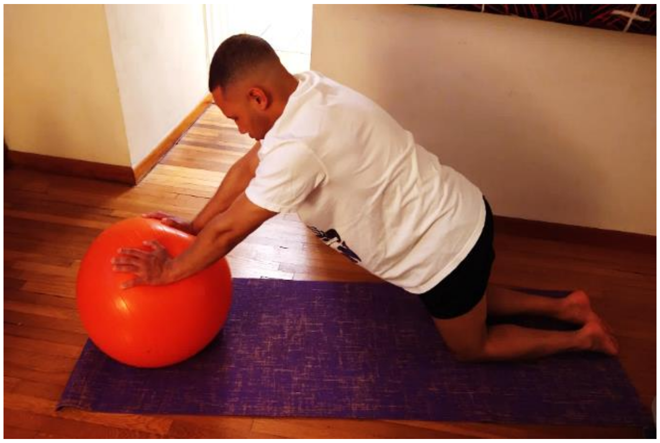

Modified from: ©A Forsberg, Y Nilsagård. This programme was developed in a research study, Group balance training for persons with multiple sclerosis". Reference. Nilsagård Y, von Koch L, Nilsson M, Forsberg A. Balance exercise program reduced falls in people with multiple sclerosis: a single group pretest, posttest trial. Archives of Physical Medicine and Rehabilitation, e-pub 6 juli 2014. Used with permission.

5. Choose ONE of the three exercises below (Level 2, Level 3 or Level 4). Repeat the exercise for 30 seconds, then take a break for 10-20 seconds.[1]

   Level 2: Slide your foot straight back on the floor, keeping it in line with your body. Then slide it back to the starting position. Repeat with the other side if possible.

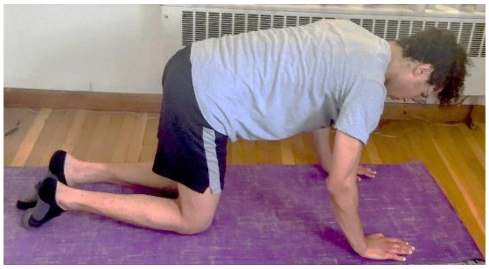
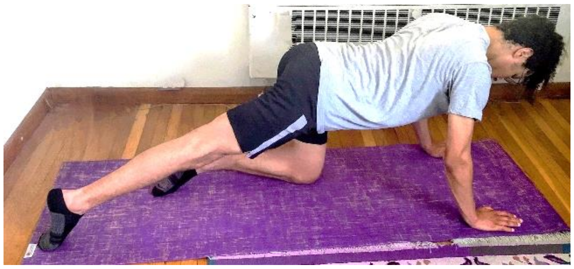

   Level 3: Slide your foot straight back on the floor, keeping it in line with your body. Next lift your leg off the floor for 1-2 seconds. Then slide it back to the starting position. Repeat with the other side if possible.

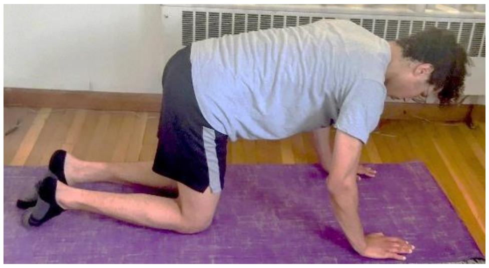
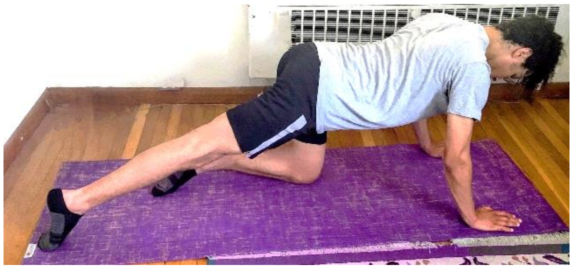
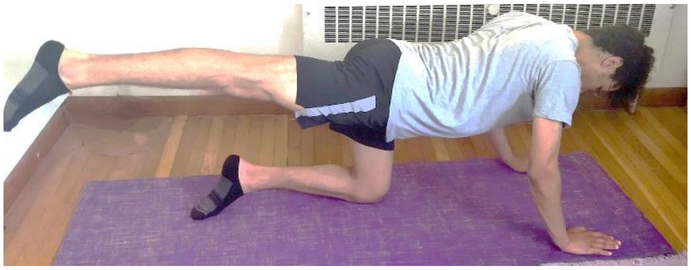

   Level 4: Lift one leg and the opposite arm slowly off the floor. Hold for 5 seconds. Repeat with the other side if possible.

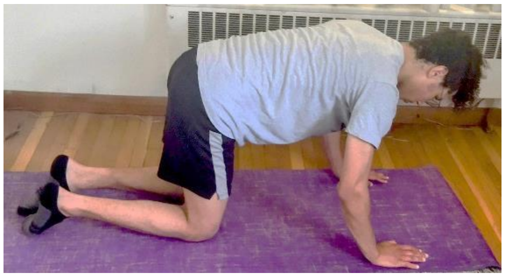
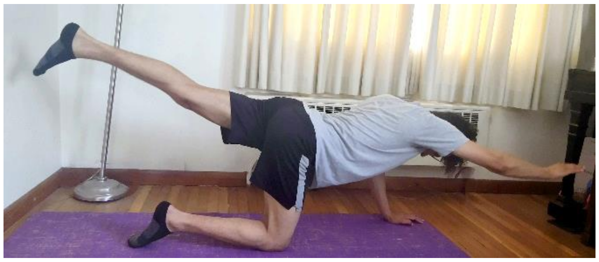

Modified from: ©A Forsberg, Y Nilsagård. This programme was developed in a research study, Group balance training for persons with multiple sclerosis". Reference. Nilsagård Y, von Koch L, Nilsson M, Forsberg A. Balance exercise program reduced falls in people with multiple sclerosis: a single group pretest, posttest trial. Archives of Physical Medicine and Rehabilitation, e-pub 6 juli 2014. Used with permission.

GROUP TWO: DUAL-TASK EXERCISES

1. Tap a balloon on your own, or tap it back and forth with a partner. Try to stand up and sit down as you are tapping. Continue for 30 seconds, then rest for 10-20 seconds.

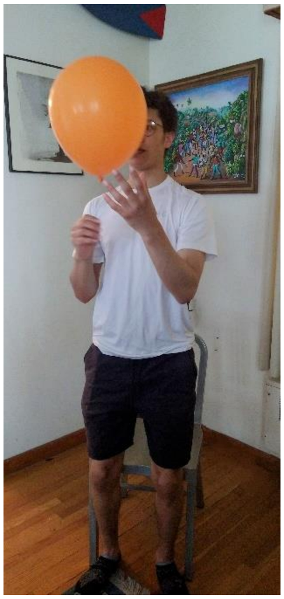

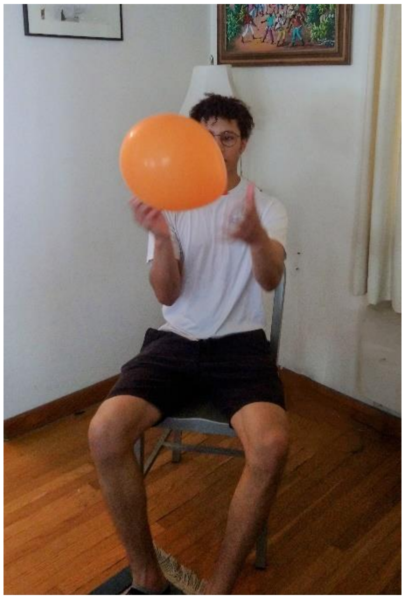

2. Repeat, using a racket or cricket bat to tap the balloon. Continue for 30 seconds, then rest for 10-20 seconds.

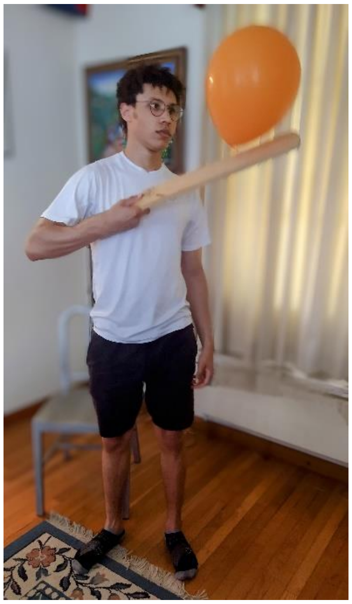

Modified from: ©A Forsberg, Y Nilsagård. This programme was developed in a research study, Group balance training for persons with multiple sclerosis". Reference. Nilsagård Y, von Koch L, Nilsson M, Forsberg A. Balance exercise program reduced falls in people with multiple sclerosis: a single group pretest, posttest trial. Archives of Physical Medicine and Rehabilitation, e-pub 6 juli 2014. Used with permission.

3. Stand up and sit down 5 times, while holding a tray with unstable items on it, such as some balls or cups. Rest for 10-20 seconds after you complete the task. If this is too difficult, start with one item on the tray, or use a small tray you can hold with one hand.

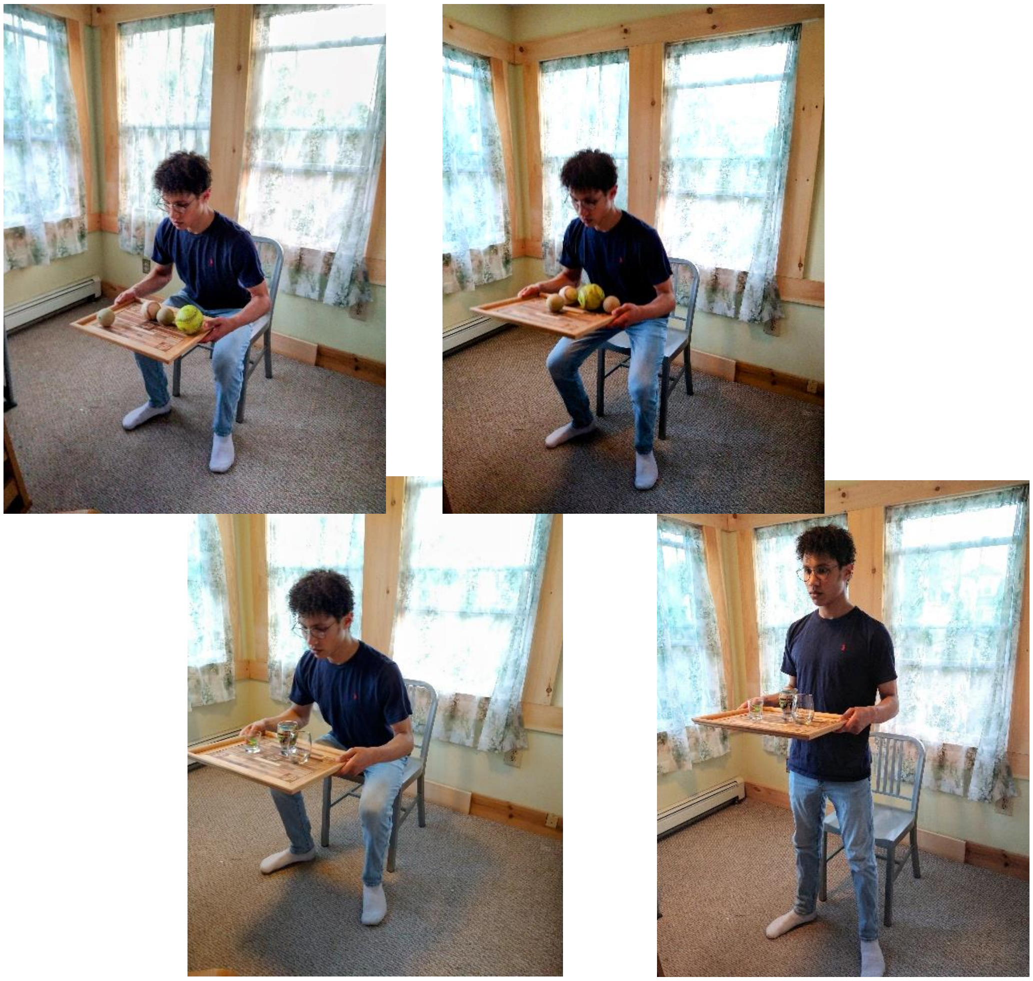

4. Pick up objects from the floor. Continue for 30 seconds, then rest for 10-20 seconds. Use larger objects if you are having difficulty bending forward.

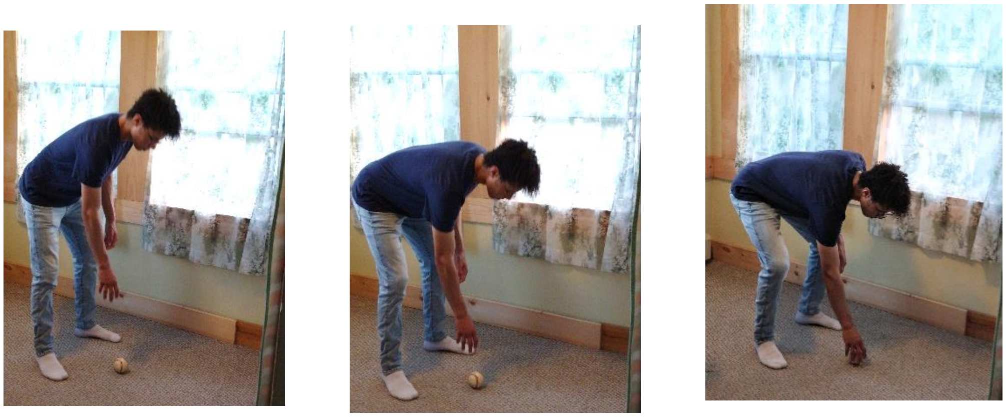

Modified from: ©A Forsberg, Y Nilsagård. This programme was developed in a research study, Group balance training for persons with multiple sclerosis". Reference. Nilsagård Y, von Koch L, Nilsson M, Forsberg A. Balance exercise program reduced falls in people with multiple sclerosis: a single group pretest, posttest trial. Archives of Physical Medicine and Rehabilitation, e-pub 6 juli 2014. Used with permission.

5. Carry the tray with items on it, and step over obstacles on the floor.   Place the tray on a table, then pick up a bag with items in it and carry the bag back to where you started, walking around the obstacles. Continue for 30 seconds, then rest for 10-20 seconds.

   If this is too difficult, you may start by carrying the bag in both directions. You can walk around the obstacles instead of stepping over them.

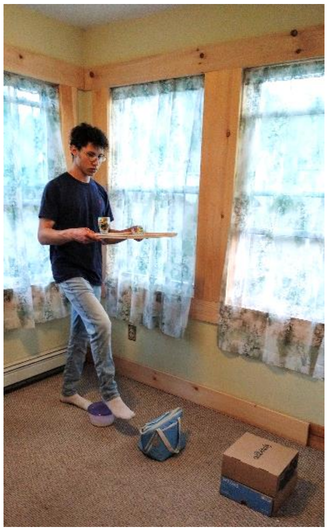
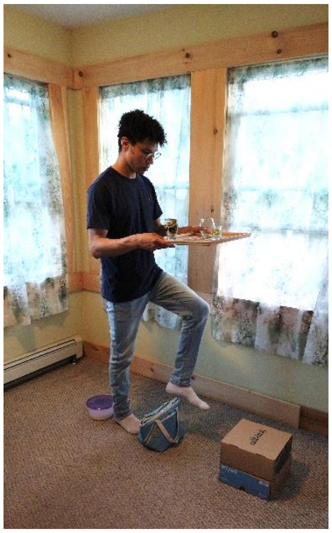
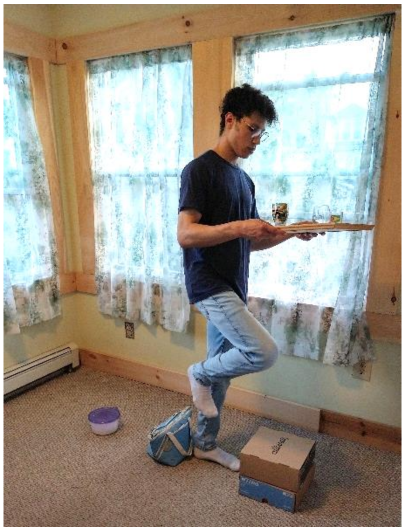
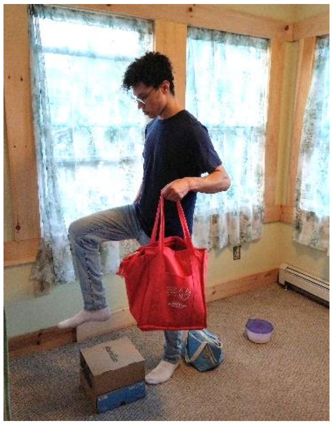
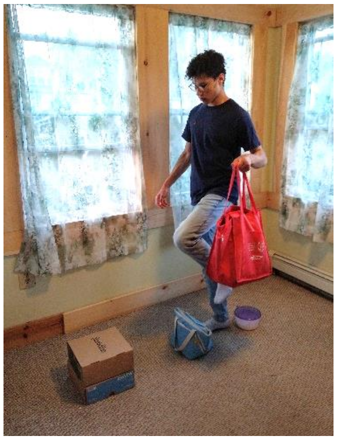
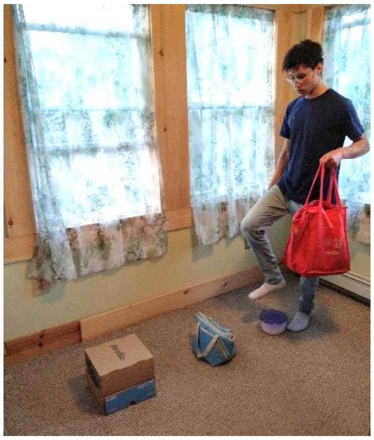

Modified from: ©A Forsberg, Y Nilsagård. This programme was developed in a research study, Group balance training for persons with multiple sclerosis". Reference. Nilsagård Y, von Koch L, Nilsson M, Forsberg A. Balance exercise program reduced falls in people with multiple sclerosis: a single group pretest, posttest trial. Archives of Physical Medicine and Rehabilitation, e-pub 6 juli 2014.   Used with permission.

6. Try to walk in a straight line with turn your head left to right, then looking up and down. Try to walk for 30 seconds, turning around as needed. Rest for 10-20 sec.

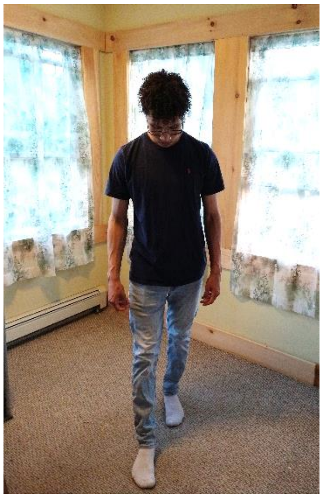
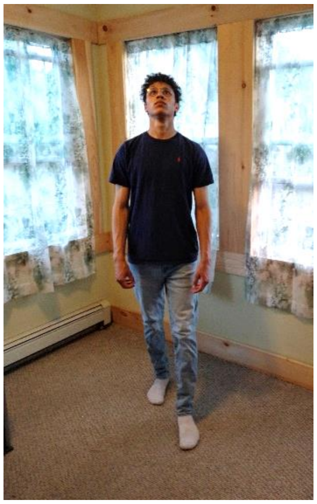
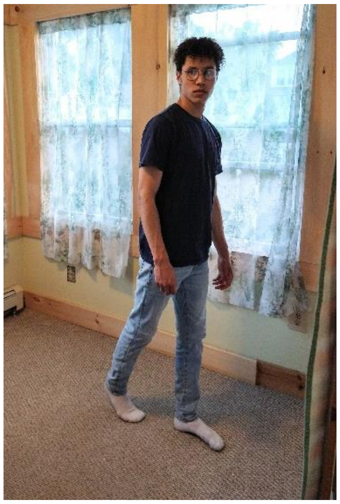
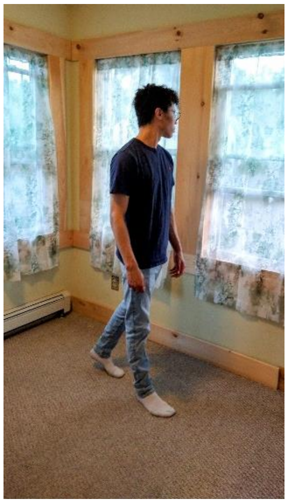

7. Try to walk continuously while listing the days of the week and the months of the year, or while listing items (such as fruits or vegetables) or counting backwards. Try to walk for 30 seconds, turning around as needed. Rest for 10-20 sec.

8. Stand up from a chair, with your feet close together. Repeat five time. Then stand up with one foot in front of the other. Repeat 5 times.

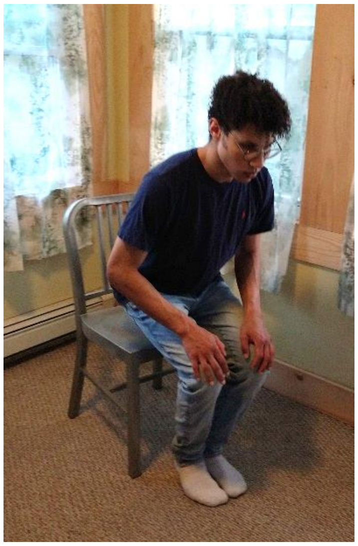
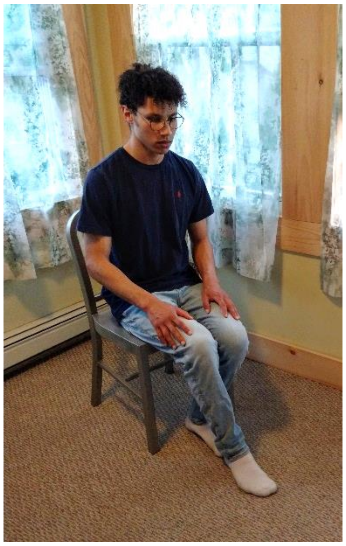
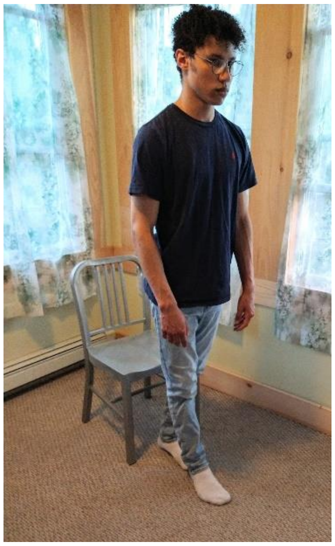

Modified from: ©A Forsberg, Y Nilsagård. This programme was developed in a research study, Group balance training for persons with multiple sclerosis". Reference. Nilsagård Y, von Koch L, Nilsson M, Forsberg A. Balance exercise program reduced falls in people with multiple sclerosis: a single group pretest, posttest trial. Archives of Physical Medicine and Rehabilitation, e-pub 6 juli 2014. Used with permission.

9. Walk forward, taking big steps and swinging your arms. Continue for 30 seconds, then rest for 10-20 seconds.

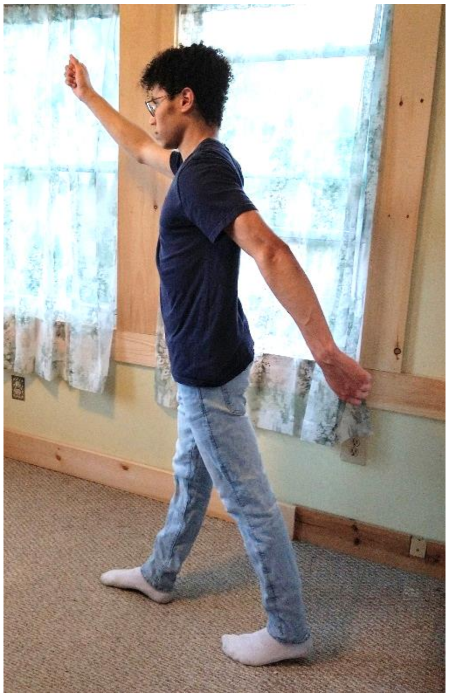

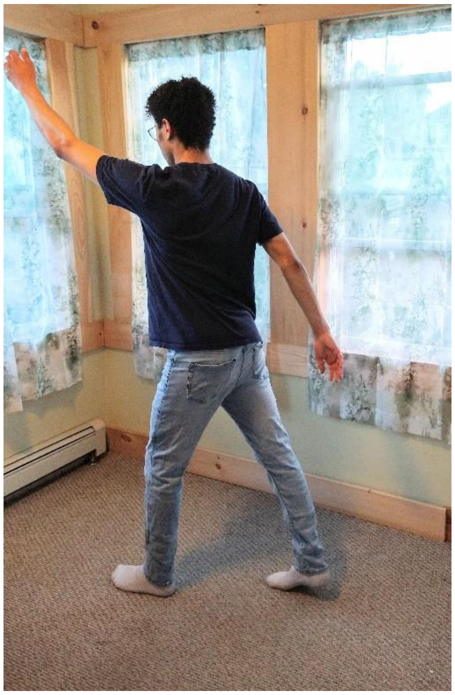

10. Walk backwards. Continue for 30 seconds, then rest for 10-20 seconds.

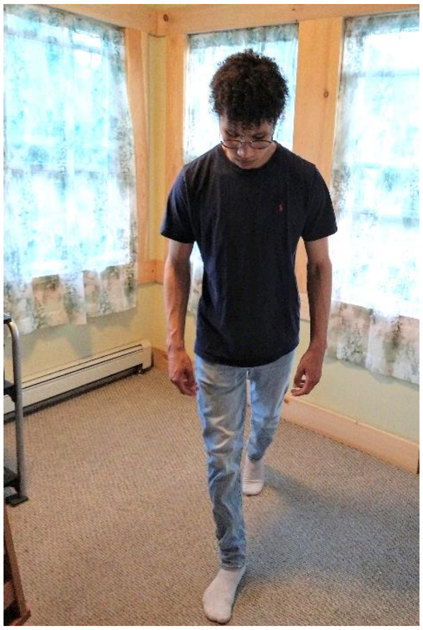

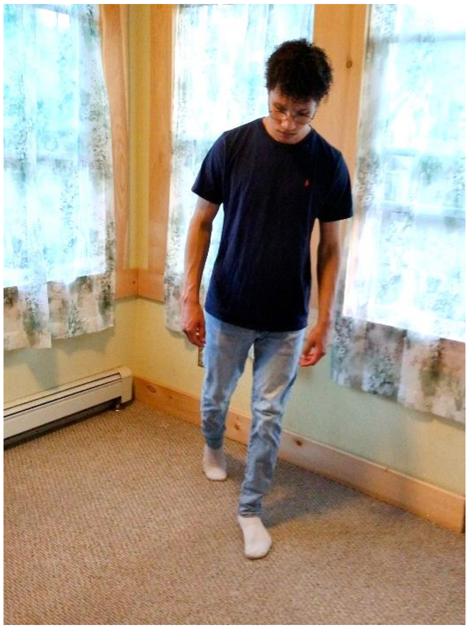

Modified from: ©A Forsberg, Y Nilsagård. This programme was developed in a research study, Group balance training for persons with multiple sclerosis”. Reference. Nilsagård Y, von Koch L, Nilsson M, Forsberg A. Balance exercise program reduced falls in people with multiple sclerosis: a single group pretest, posttest trial. Archives of Physical Medicine and Rehabilitation, e-pub 6 juli 2014.   Used with permission.

11. Walk sideways for three steps. As you step, cross one leg in front of the other. When you return, cross the opposite leg in front. Repeat for 30 seconds, then rest for 10-20 seconds.

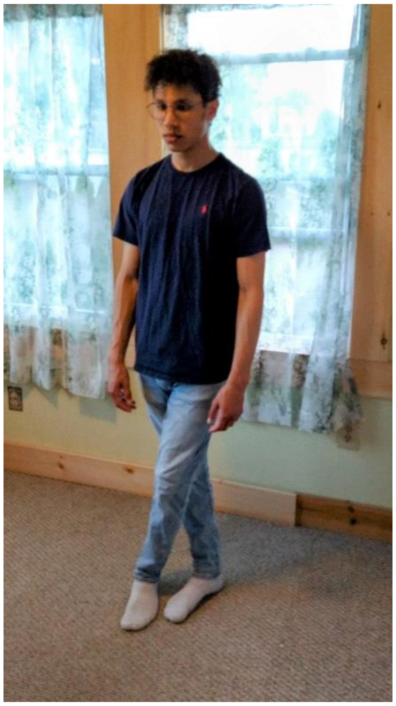

3

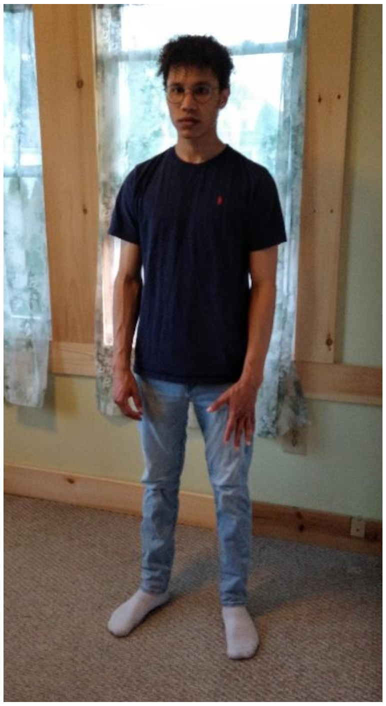

2

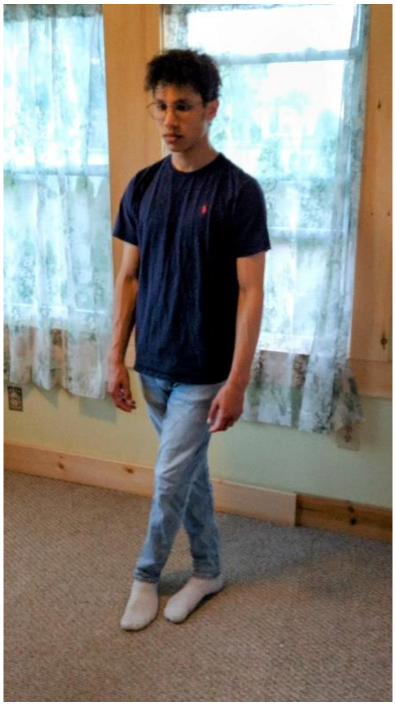

1

Modified from: ©A Forsberg, Y Nilsagård. This programme was developed in a research study, Group balance training for persons with multiple sclerosis". Reference. Nilsagård Y, von Koch L, Nilsson M, Forsberg A. Balance exercise program reduced falls in people with multiple sclerosis: a single group pretest, posttest trial. Archives of Physical Medicine and Rehabilitation, e-pub 6 juli 2014. Used with permission.

PART THREE: USING SENSORY STRATEGIES

1. Walk on an uneven surface, such as a yoga mat with small items or folded towels underneath. Walk back and forth for 30 seconds to 1 minute, then rest for 10-20 seconds.

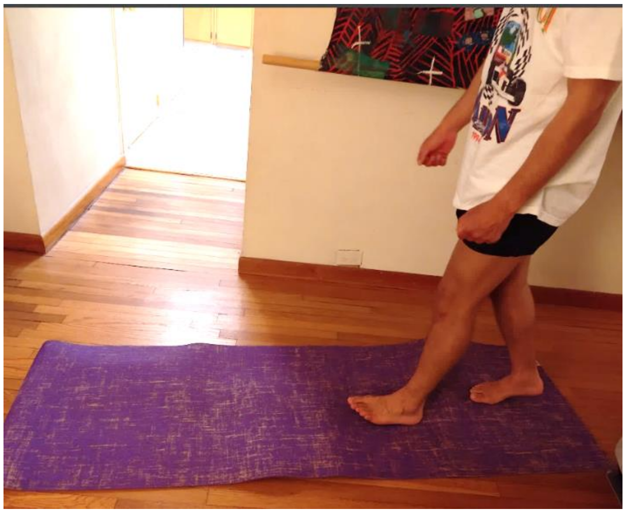

2. Move between standing up and sitting down, with your eyes closed. Repeat 5 times.

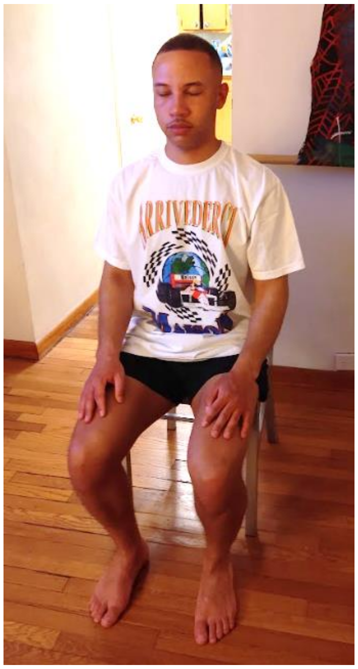

Modified from: ©A Forsberg, Y Nilsagård. This programme was developed in a research study, Group balance training for persons with multiple sclerosis". Reference. Nilsagård Y, von Koch L, Nilsson M, Forsberg A. Balance exercise program reduced falls in people with multiple sclerosis: a single group pretest, posttest trial. Archives of Physical Medicine and Rehabilitation, e-pub 6 juli 2014.   Used with permission.

3. Move between standing up and sitting down, with your feet shoulder width apart while standing on a cushion. Repeat 5 times with eyes open, then 5 times with eyes closed.

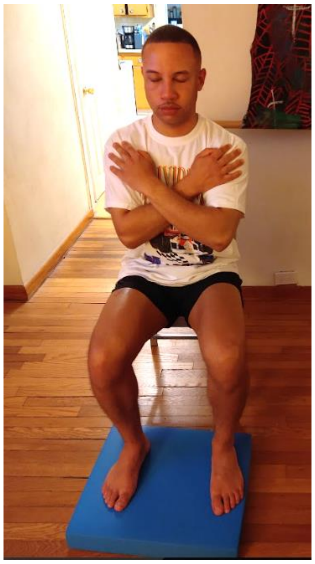

4. Move between standing up and sitting down, with your feet together while standing on a cushion. Repeat 5 times with eyes open, then 5 times with eyes closed.

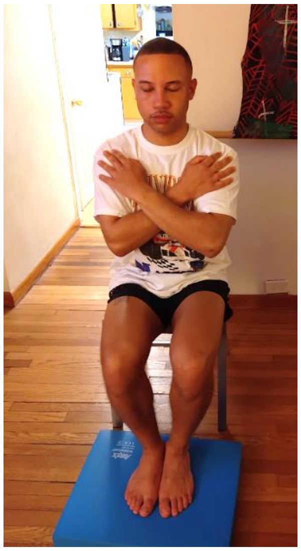

Modified from: ©A Forsberg, Y Nilsagård. This programme was developed in a research study, Group balance training for persons with multiple sclerosis”. Reference. Nilsagård Y, von Koch L, Nilsson M, Forsberg A. Balance exercise program reduced falls in people with multiple sclerosis: a single group pretest, posttest trial. Archives of Physical Medicine and Rehabilitation, e-pub 6 juli 2014.   Used with permission.

5. Sit on a therapy ball and hold a tray with small balls or cups. Practice standing up and sitting back down 5 times.

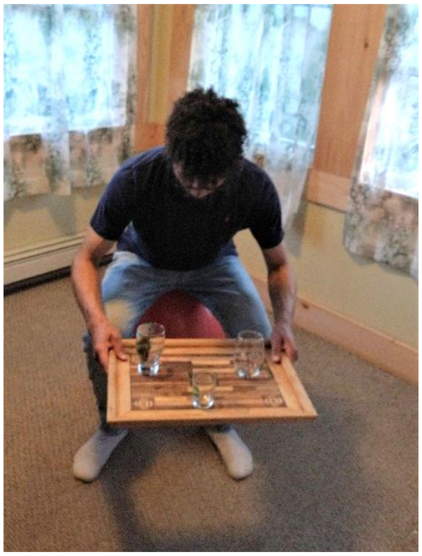

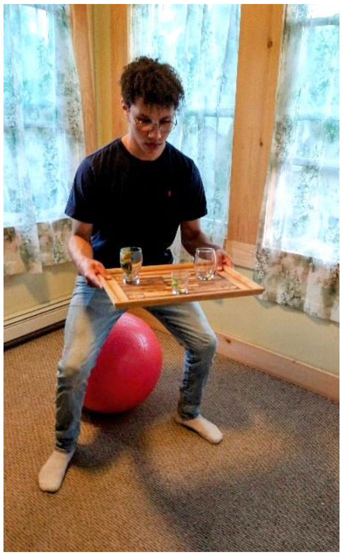

Modified from: ©A Forsberg, Y Nilsagård. This programme was developed in a research study, Group balance training for persons with multiple sclerosis". Reference. Nilsagård Y, von Koch L, Nilsson M, Forsberg A. Balance exercise program reduced falls in people with multiple sclerosis: a single group pretest, posttest trial. Archives of Physical Medicine and Rehabilitation, e-pub 6 juli 2014. Used with permission.

6. Perform different standing exercises while on a cushion or Bosu ball (half ball). Make sure you are near a chair or a surface you can use for support. Exercises can include: standing with weight on both legs, turning to look behind you, tapping a balloon, or playing catch with a ball. Try to perform each exercise for 30 seconds, resting for 10-20 seconds between exercises.

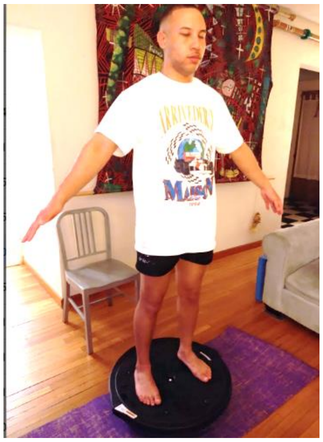

Balance on Bosu

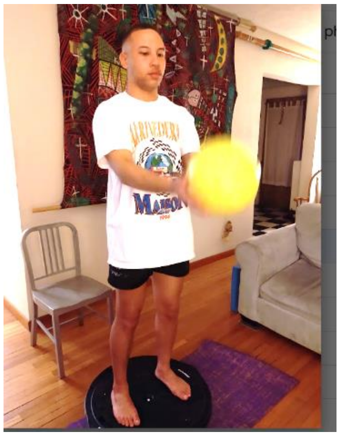

Catching ball on Bosu

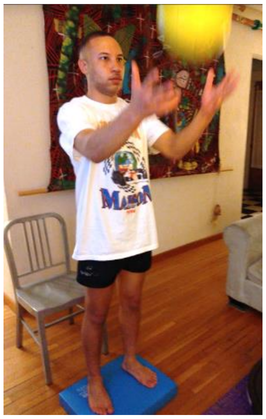

Catching ball on foam

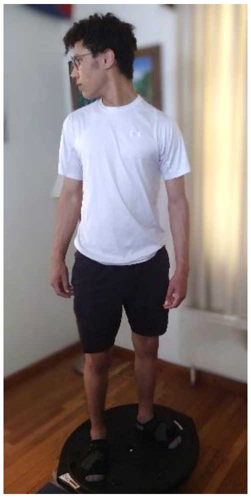

Looking back on Bosu

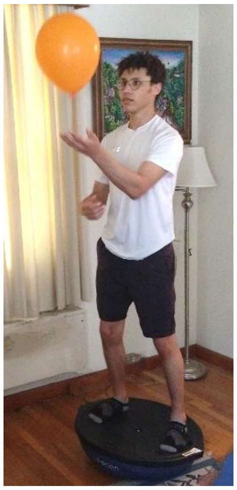

Tapping balloon on Bosu

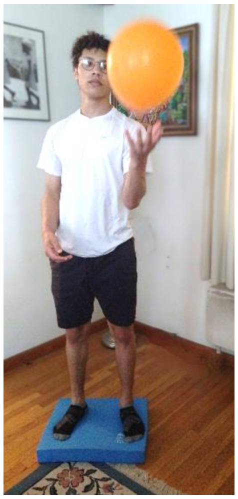

Tapping balloon on foam

Modified from: ©A Forsberg, Y Nilsagård. This programme was developed in a research study, Group balance training for persons with multiple sclerosis". Reference. Nilsagård Y, von Koch L, Nilsson M, Forsberg A. Balance exercise program reduced falls in people with multiple sclerosis: a single group pretest, posttest trial. Archives of Physical Medicine and Rehabilitation, e-pub 6 juli 2014. Used with permission.

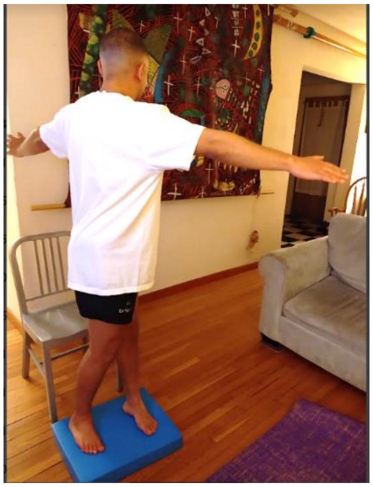

Trunk rotation on foam - feet shoulder width apart

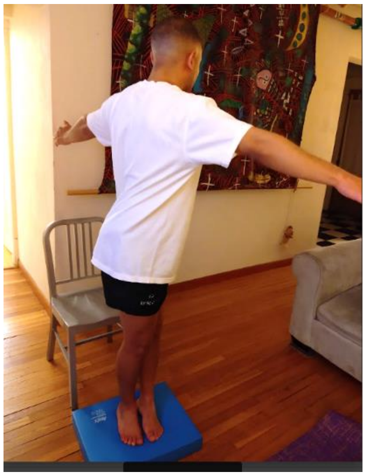

Trunk rotation on foam - feet together

Modified from: ©A Forsberg, Y Nilsagård. This programme was developed in a research study, Group balance training for persons with multiple sclerosis". Reference. Nilsagård Y, von Koch L, Nilsson M, Forsberg A. Balance exercise program reduced falls in people with multiple sclerosis: a single group pretest, posttest trial. Archives of Physical Medicine and Rehabilitation, e-pub 6 juli 2014.   Used with permission.

T'ai Chi exercises – to work on breathing, shifting weight, lower extremity strength

1) **Chi Breathing**

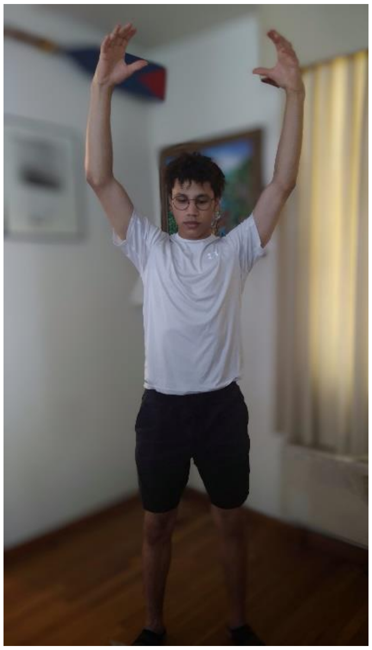

As you inhale, lifting your arms overhead, and straighten your knees.

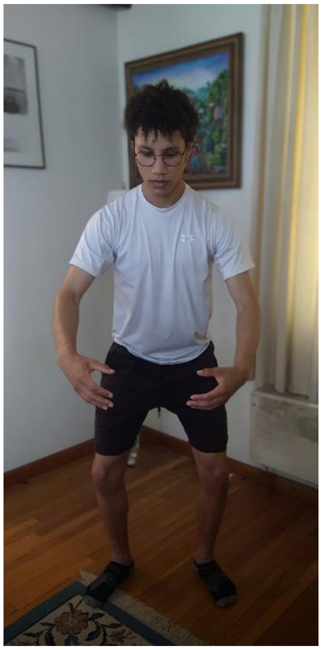

Exhale, bringing your hands down toward your waist, and bend your knees slightly.

Repeat 3-5 times.

2) Painting with Light

As you inhale, lift your arms out in front of you, keeping elbows straight, and fingers pointed toward the floor.

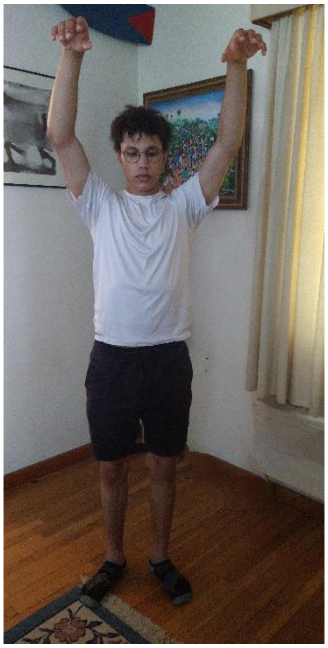

As you exhale, point your fingers toward the ceiling, bend your elbows, and bend your knees slightly.

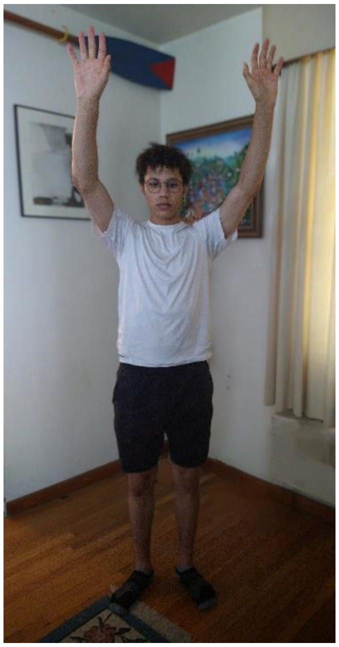

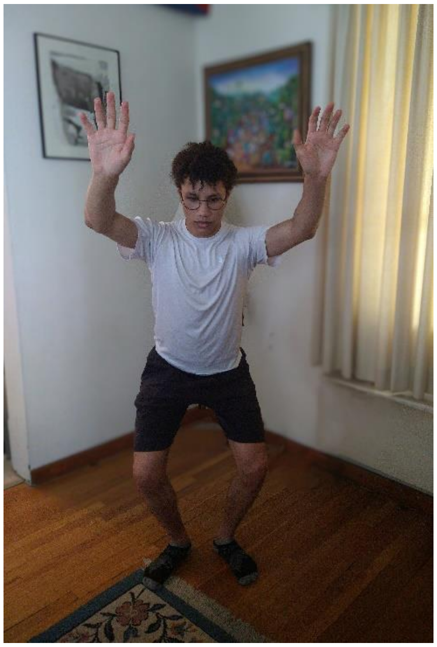

Repeat 3-5 times.

3) Push Waters to the Side

Stand with your feet shoulder width apart, and your knees slightly bent.

Shift your weight so it's mostly on your right foot. Your arms should be out in front, with your left arm higher than the right, both hands facing left.

Tighten your core muscles, then slowly shift your weight onto your left foot as your move both arms to the left, like your pushing water in a pool from right to left.

Once your weight is on the left, switch your arm position: your right arm should be higher than the left, and both hands should face right.

Tighten your core muscles, then slowly shift your weight onto your right foot as you move both arms to the right.

Repeat 3-5 times.

1

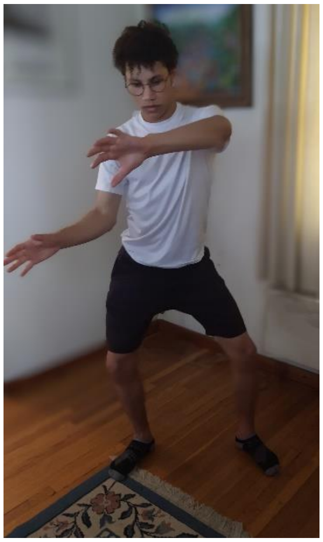

2

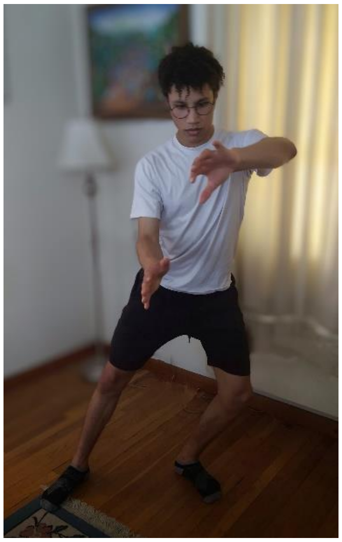

3

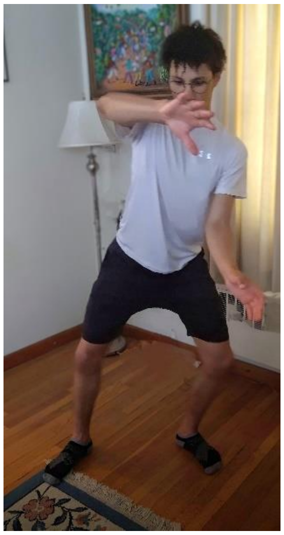

4

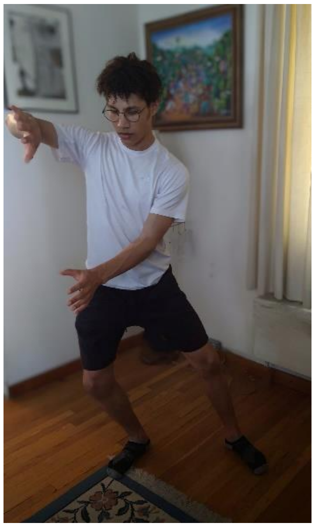

Supplement: 2023-0439_R2_Supplementary_Material_1_pzae107 [file 2023-0439_r2_supplementary_material_1_pzae107.pdf]
