# Supplementary material for: A Falls Prevention Program for People After Stroke in Guyana: An International Collaboration
Source: Phys Ther. 2024 Aug 7;104(10):pzae107. doi: 10.1093/ptj/pzae107 (PMC11523612; doi:10.1093/ptj/pzae107)
Supplement: 2023-0439_R2_Supplementary_Material_2_pzae107 [file 2023-0439_r2_supplementary_material_2_pzae107.pdf]

Supplementary Material 2. Pretest and Posttest Questionnaires

Research Project Questionnaire

Title of Research Project: Exercises to Reduce Falls in Persons Post-Stroke

1. What is your age?

30-35 ☐ 35-40 ☐ 40-45 ☐ 45-50 ☐ 50-55 ☐ 55-60 ☐ 60-65 ☐ 65-70 ☐ 70-75 ☐

2. What is your gender?

Male ☐ Female ☐

3. What is your race?

African ☐ Amerindian ☐ East-Indian ☐ Chinese ☐ Mixed-Race ☐

4. What is your occupation?

____

5. When did you have your stroke?

Less than a week ago ☐ One month ago ☐

Over three months ago ☐ Over six months ago ☐

____

6. Was this your first stroke? If no, kindly indicate how many you've had.

Yes ☐ No ☐

If no: ___________________________

7. Have you fallen since the stroke?

Yes ☐ No ☐

8. If yes, kindly indicate how many times and when the fall occurred.

Once ☐ Twice ☐ Over 3 times ☐

Less than a week ago ☐ One month ago ☐ Three months ago ☐

9. If you have fallen, where did it occur?

Inside your home ☐

Outside your home or yard ☐

In the community ☐

10. How concerned or not concerned are you about falling over?

Very concerned ☐

Fairly concerned ☐

Not very concerned ☐

Not concerned at all ☐

Don’t know ☐

11. Do you take more than 3 prescription medications each day?

Yes ☐ No ☐

12. Do you take medications?

- To help you sleep? ☐
- To help control mood (e.g. anxiety, depression)? ☐
- To help prevent seizures or control heart rhythm? ☐
- To control pain? ☐
- To control blood pressure? ☐

13. Do you have vision problems? Such as blurriness, difficulty seeing to the side, different depths, distances or that you are sensitive to changing light?

Yes ☐ No ☐

14. Do you have numbness or loss of sensation in your feet?

Yes ☐ No ☐

15. Are you fearful of falling, such that it limits your willingness to do activities that you enjoy?

Yes ☐ No ☐

Fall Prevention Research

Follow-up Questionnaire

1. Have you had any since the starting of the program?
2. Where did you have these falls?
3. Did you like the exercises?
    a. Are you still doing the exercises at home?
    b. If No, Why not?
4. Have you been walking around your community?
    a. Any falls when doing so?
    b. Walking alone or with a family member?
5. How concerned or not concerned are you about falling over?
    - Very Concerned
    - Fairly Concerned
    - Not very concerned
    - Not concerned at all
    - Don’t know
6. Are you fearful of falling, such that it limits your willingness to do activities you enjoy?
7. Are you getting support from your family when doing the exercise program at home?
8. Have you been able to go back to your daily activities of living since starting the program?
